# Supplementary material for: Optimization of Revision Hip Arthroplasty Workflow by Means of Detailed Pre-Surgical Planning Using Computed Tomography Data, Open-Source Software and Three-Dimensional-Printed Models
Source: Diagnostics (Basel). 2023 Jul 28;13(15):2516. doi: 10.3390/diagnostics13152516 (PMC10417331; doi:10.3390/diagnostics13152516)
Supplement: Supplementary file 1 [file diagnostics-13-02516-s001.zip › diagnostics-2476253-supplementary.pdf]

Supplementary Materials

Optimization of revision hip arthroplasty workflow by means of detailed pre-surgical planning using CT data, open-source software and 3D printed models

Krzysztof Andrzejewski [1], Marcin Domżalski [1], Piotr Komorowski [3], Jan Poszepczyński [1], Bożena Rokita [4] and Marcin Elgalal [2,*]

[1] Veteran's Memorial Hospital Medical University of Lodz, Zeromskiego 113, 90-549 Lodz, Poland; kj.andrzejewski@outlook.com; janek24061982@tlen.pl; marcin.domzalski@umed.lodz.pl

[2] Second Department of Radiology and Diagnostic Imaging, Medical University of Lodz, Pomorska 251, 92-213 Lodz, Poland; marcin.elgalal@umed.lodz.pl

[3] Division of Biophysics, Institute of Materials Science, Lodz University of Technology, Stefanowskiego 1/15, 90-924 Lodz, Poland; piotr.jerzy.komorowski@gmail.com

[4] Institute of Applied Radiation Chemistry, Faculty of Chemistry, Lodz University of Technology, Wroblewskiego 15, 93-590 Lodz, Poland; bozena.rokita@p.lodz.pl

*Correspondence: marcin.elgalal@umed.lodz.pl; Tel.: +48 42 201 42 06

Table S1. Centre of rotation position (mean ± SD, n=10).

	Preoperative distance [mm]	3D planning distance [mm]	Postoperative distance [mm]
X-axis (Coronal plane)	86.3 ± 8.7	86.0 ± 7.8	87.5 ± 8.6
Y-axis (Coronal plane)	71.7 ± 9.6	71.3 ± 11.8	66.6 ± 10.5
Z-axis (Axial plane)	52.2 ± 4.8	51.8 ± 2.3	53.7 ± 4.5

Table S2. Correlation of presurgical 3D planning and post-operative COR position measurements, acetabular and inclination angles (mean ± SD, n=10, $p < 0.05$, *- statistical significant, ns- no significant).

	3D planning results mm / deg.	Postoperative results mm / deg.	Pearson r	*p* value
X-axis (Coronal plane)	86.0 ± 7.8	87.5 ± 8.6	0.6720 (ns)	0.0982
Y-axis (Coronal plane)	71.3 ± 11.8	66.6 ± 10.5	0.9438 *	0.0014
Z-axis (Axial plane)	51.8 ± 2.3	53.7 ± 4.5	0.8829 *	0.0084
Inclination angle	47.7 ± 5.6	51.6 ± 5.3	-0.4042 (ns)	0.3685
Anteversion angle	17.3 ± 4.1	15.4 ± 5.2	-0.4782 (ns)	0.2777

Table S3. Anteversion and angle of inclination values (mean ± SD, n=10).

Parameters	Preoperative [deg.]	3D planning [deg.]	Postoperative [deg.]
Inclination angle	55.7 ± 15.4	47.7 ± 5.6	51.6 ± 5.3
Anteversion angle	21.1 ± 9.7	17.3 ± 4.1	15.4 ± 5.2

Krzysztof Andrzejewski et al. Correspondence: marcin.elgalal@umed.lodz.pl; Tel.: +48 42 201 42 06
